# Diabetic Ketoacidosis Precipitated by COVID-19 in Patients Without Respiratory Symptoms: Case Reports

**DOI:** 10.7759/cureus.10031

**Published:** 2020-08-25

**Authors:** Jose L Meza, Abel Triana, Ivan De Avila, Gaspar Del Rio-Pertuz, Diego Viasus

**Affiliations:** 1 Emergency, Hospital Universidad del Norte, Universidad del Norte, Barranquilla, COL; 2 Internal Medicine, Texas Tech University Health Sciences Center, Lubbock, USA; 3 Infectious Diseases, Hospital Universidad del Norte, Universidad del Norte, Barranquilla, COL

**Keywords:** covid-19, ketoacidosis, diabetes, coinfection, mortality, sars-cov-2

## Abstract

Worse outcomes of coronavirus disease 2019 (COVID-19) have been documented in older patients with comorbidities, especially in those with diabetes mellitus (DM). However, the clinical picture and risk factors of COVID-19 in DM is still emerging. Here, we report four cases of severe COVID-19 patients with acute diabetic ketoacidosis (DKA) without respiratory symptoms, with viral and bacterial coinfection, and poor clinical outcomes. Higher monitoring of patients with DM and COVID-19 is advised, as well as rapid and accurate diagnostic tests and treatment.

## Introduction

In December 2019, an acute respiratory illness of unknown origin occurred in Wuhan, China. The causal microorganism, which was named severe acute respiratory syndrome coronavirus 2 (SARS-CoV-2) [1], has spread worldwide causing a pandemic. The typical clinical manifestations include fever, dry cough, sore throat, myalgia, fatigue, headache, nausea, and diarrhea. However, new clinical features or complications have been reported such as neurological or cutaneous manifestations [2-3]. Diabetes mellitus (DM) has been one of the most common comorbidities associated with patients having coronavirus disease 2019 (COVID-19) with a prevalence ranging from 1.6% to 20% [4]. Patients with pre-existing DM seem to have a higher risk of developing severe metabolic complications, requiring ICU admissions, and death [5]. However, clinical picture and risk factors in DM are emerging and the diabetogenic characteristic of SARS-CoV-2 is still under investigation.

In this report, we highlight the important issues related to four cases of severe COVID-19 presenting with diabetic ketoacidosis (DKA). This report was approved by the Institutional Review Board.

## Case presentation

Case 1

**Figure 1:**
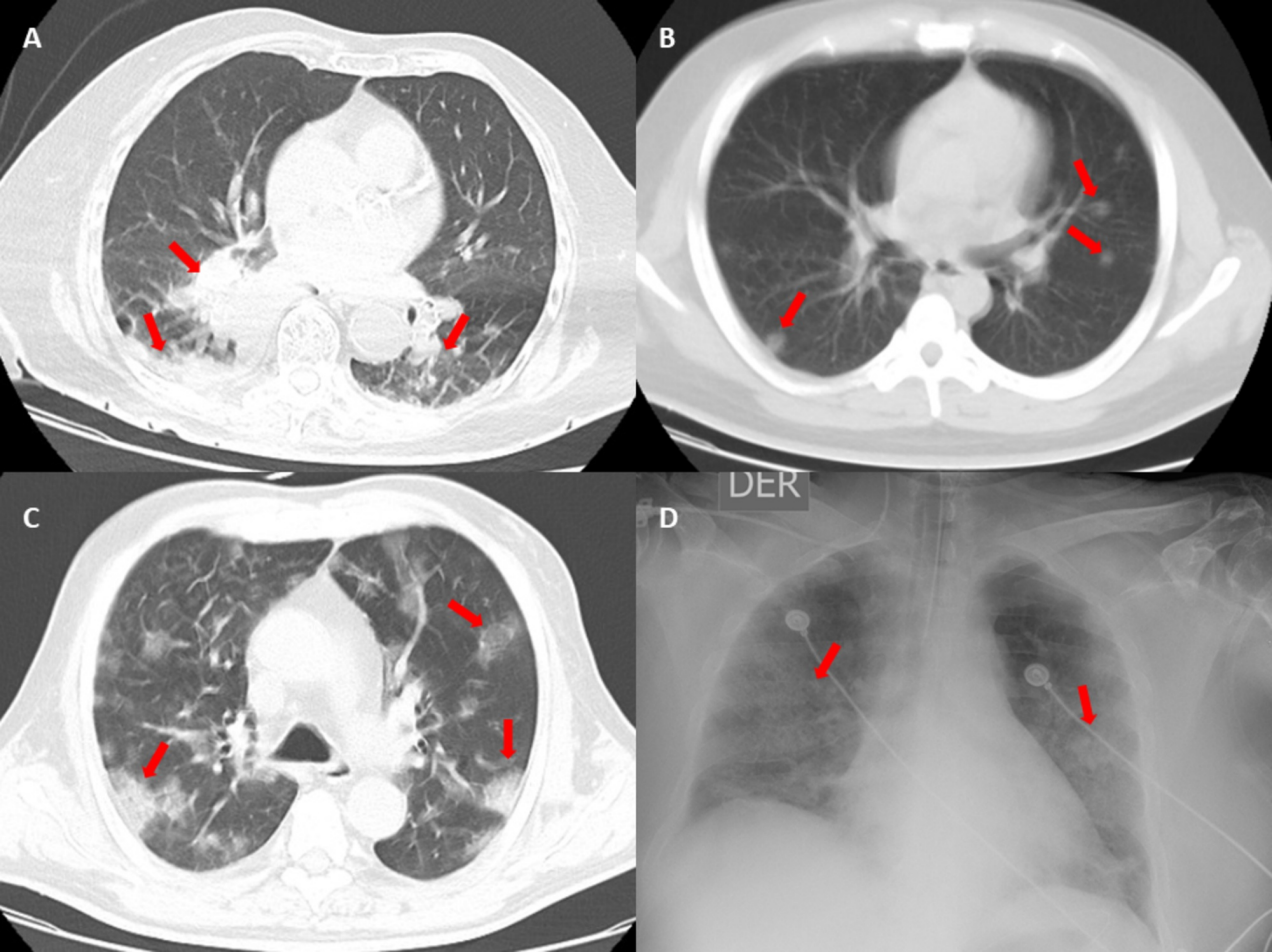
A: Chest CT at admission from Case 1. A mass that infiltrates the bronchi for the basal segments of the right lower lobe, as well as the presence of right basal pleural effusion and ground-glass consolidation. B: Chest CT at admission from Case 2. Multiple bilateral ground-glass opacities. C: Chest CT at admission from Case 3. Multiple bilateral crazy-paving consolidations. D: Chest radiograph at admission from Case 4. Multiple bilateral pulmonary consolidations.

An 87-year-old female was admitted to the ED suffering from four days of polydipsia and high capillary blood glucose levels, two days of fever, somnolence, and a recent fall. She had a history of type 2 diabetes mellitus (T2DM). The physical examination revealed disorientation and drowsiness. At admission, plasma glucose and hemoglobin A1C (HbA1C) were 752 mg/dL and 12.4%, respectively. Acute kidney injury (AKI) and DKA were diagnosed based on blood chemistry (Table 1). A chest CT showed bilateral ground-glass opacity (Figure 1A).

**Table 1:** Clinical laboratory results at hospital admission. *Not corrected sodium F, female; M, male; HbA1C, hemoglobin A1C; PaCO2, partial pressure of arterial carbon dioxide; HCO3, bicarbonate; PaO2, partial pressure of arterial oxygen

Measure	Reference range	Case 1	Case 2	Case 3	Case 4
White cell count (per μL)	4000-11,000	17,170	19,540	21,830	6,630
Absolute neutrophil count (per μL)	2000-8250	15,140	16,1600	20,310	5,608
Absolute lymphocyte count (per μL)	900-5200	660	1,480	710	842
Platelet count (per μL)	150,000-450,000	199,000	484,000	311,000	261,000
Hematocrit (%)	M: 42-50, F: 37-47	39.7	58.6	47.8	39.4
HbA1C (%)	<7	12.4	18	14	Not performed
Sodium (mEq/L) *	136-145	138.3	138.1	127.8	132
Potassium (mEq/L)	3.5-5	4.50	5.89	7.18	5.15
Chloride (mEq/L)	98-106	100	101	89	97.8
Anion gap (mmol/L)	7-13	37.5	39.8	43.6	31.1
Blood urea nitrogen (mg/dL)	5-25	48.13	21.03	42.06	32.40
Creatinine (mg/dL)	M: 0.5-1.30, F: 0.4-1.2	2.86	1.41	3.53	2.19
D-Dimer (mg/L)	Less than 0.5	6.70	1.60	2.96	0.45
Ferritin (ng/ml)	M: 24-336 ng/mL, F: 11-307 ng/mL	461.5	>1650	1234	1291
Lactate dehydrogenase (U/L)	80-225	335	427	330	670
Plasma glucose (mg/dL)	70-99	752	549	933	727
pH (arterial)	7.350-7.450	7.039	7.112	7.032	7.008
paO2 (mmHg)	75-100	86.7	91.3	103.3	61
HCO3 (mmHg)	21-23	5.3	3.2	2.4	9
paCO2 (mmHg)	35-45	20.1	10.3	9.2	36

On day two, a rapid decline of the respiratory status resulted in oxygen supplementation and mechanical ventilation. Vasopressors were also started. On day five the patient required renal replacement therapy due to worsening AKI and died. She was positive for adenovirus, influenza type B, and SARS-CoV-2.

Case 2

An overweight 24-year-old male was admitted to the ED with 13 days of weakness, polydipsia, polyuria, nausea, and vomiting. Physical examination revealed somnolence, tachycardia, tachypnea, and Kussmaul breathing. Upon admission blood chemistry revealed plasma glucose of 549 mg/dL, HbA1c of 18%, AKI and DKA criteria (Table 1). A chest CT showed bilateral peripheral ground-glass pattern (Figure 1B). Bicarbonate infusion was started due to refractory severe metabolic acidosis. Respiratory status declined rapidly which led to the need for mechanical ventilation. On hospital day four, blood culture reported Salmonella enteritis. Due to refractory metabolic acidosis, renal replacement therapy was performed. The patient was progressively improving, extubated, and discharged on day 10. He was positive for influenza type A and SARS-CoV-2.

Case 3

A 66-year-old male was admitted to the ED complaining of four days of diarrhea, vomiting, asthenia, and somnolence. He had a history of T2DM and hypertension. Physical examination revealed a disoriented patient with incoherent speech, tachycardia, tachypnea, and Kussmaul breathing. Upon admission, blood chemistry revealed plasma glucose of 933 mg/dL, HbA1c of 14%, AKI and DKA criteria (Table 1). A chest CT showed a crazy-paving pattern consolidation (Figure 1C). On hospital day two, oxygen supplementation was started, and blood cultures showed Staphylococcus hominis. On hospital day three, the patient developed psychomotor agitation and a rapid decline in respiratory status which derived in mechanical ventilation and died on this day. He was positive for SARS-CoV-2.

Case 4

A 68-year-old male presented to the ED with one day of general weakness, associated with high blood glucose levels. He had a history of T2DM. Physical examination revealed tachycardia and hypertension. Upon admission, plasma glucose was 727 mg/dL, AKI and DKA criteria were documented in blood chemistry (Table 1). A chest X-ray reported bilateral radiopacity of the lungs (Figure 1D). The patient developed altered mental status, acute respiratory distress which required mechanical ventilation. During the procedure, the patient underwent cardiac arrest. On hospital day four, the patient did not recover from respiratory failure and hemodynamic instability and died. He was positive for SARS-CoV-2.

## Discussion

Information regarding clinical characteristics, complications, and outcomes of COVID-19 is rapidly evolving as data continue to emerge throughout the world. The prevalence of DM in patients with moderate COVID-19 is nearly 10%, being higher in severe patients (17%) [4]. DM has been consistently associated with high morbidity and mortality in patients with COVID-19. In this regard, a meta-analysis documented that DM in patients with COVID-19 is related with a two-fold increase in mortality as well as severe disease, as compared to nondiabetic patients [5].

These case reports highlight important issues in DM patients with DKA. First, we documented the variability of the clinical picture of COVID-19. We have recognized patients without respiratory symptoms with pulmonary infiltrates, with severe metabolic complication, and a rapid decline in the respiratory status leading to the need for mechanical ventilation. Second, SARS-CoV-2 must be considered as a cause of metabolic decompensation in DM patients even in patients without respiratory symptoms. In this regard, adequate use of personal protective equipment should be considered in the attention of these patients until SARS-CoV-2 is ruled out. Third, bacterial coinfection should be investigated in patients with DM decompensation secondary to COVID-19. The role of coinfection in prognosis required further studies. Finally, DKA precipitated by COVID-19 is related to unpredicted worse outcomes. In the present case reports, the patients with pre-existing DM died and the patient with newly diagnosed DM progressed to AKI that required renal replacement therapy as an outpatient treatment. Therefore, DKA during the COVID-19 pandemic required rapid and accurate diagnosis tests and treatment.

## Conclusions

SARS-CoV-2 must be considered as a potential cause of metabolic decompensation in DM patients even in patients who do not have respiratory symptoms and adequate use of individual protective equipment should be considered in the attention of patients with DKA until SARS-CoV-2 is ruled out. In addition, bacterial and viral coinfection should be investigated in patients with COVID-19 and acute DKA.
